# Undernutrition Risk in Community-Living Older Adults: Post-COVID-19 Results from the 2023 U.S. National Survey of Older Americans Act Participants

**DOI:** 10.3390/nu18101619

**Published:** 2026-05-20

**Authors:** Johanna T. Dwyer, Jaime J. Gahche, Mary Beth Arensberg, Laura Borth, Shirley Chao, Judy Simon, Lydia McGrath, Anna Picard

**Affiliations:** 1 Office of Dietary Supplements, National Institutes of Health, Bethesda, MD 20892, USA; 2 Tufts Medical Center Hospital, School of Medicine, and Friedman School of Nutrition, Tufts University, Boston, MA 02111, USA; 3 Division of Abbott, Abbott Nutrition, Columbus, OH 43219, USA; 4 National Association of Nutrition and Aging Services Programs (NANASP) and Defeat Malnutrition Today (DMT), Washington, DC 20006, USA; 5 Food Policy Insights, Andover, MA 01845, USA; chao.shirley1@gmail.com; 6 Nutrition and Health Promotion Consultant, Easton, MD 21804, USA; judy.r.simon@gmail.com; 7 Brigham and Women’s Hospital, Boston, MA 02115, USA

**Keywords:** malnutrition, undernutrition, nutrition status, aged, Older Americans Act (OAA) Nutrition Services Program, community nutrition program, National Survey of Older Americans Act Participants (NSOAAP), undernutrition risk, Malnutrition Screening Tool (MST), home-delivered meals, congregate meals

## Abstract

**Background**: The Older Americans Act (OAA) home-delivered and congregate meal programs and related nutrition services are the largest federal programs in the United States (U.S.) to reduce malnutrition (undernutrition) among community-dwelling older adults. However, the prevalence of undernutrition has received little attention in the OAA programs, while many studies report the prevalence of overweight and obesity. **Objective**: We documented undernutrition risk prevalence estimates post-COVID-19 in a 2023 nationally representative survey of OAA participants in the U.S. Undernutrition risk prevalence may have been elevated among those surveyed previously in 2022 because data were collected during the COVID-19 pandemic. **Methods**: Data were obtained from the 2023 National Survey of Older Americans Act Participants (NSOAAP) (n = 4159); a cross-sectional survey of OAA participants randomly selected from a stratified sample of Area Agencies on Aging throughout the U.S. The NSOAAP included measurement of undernutrition risk, the main outcome of interest, using the Malnutrition Screening Tool (MST) that queried self-reports of unintended weight loss and decreased food intake due to poor appetite. MST scores ≥ 2 were defined as indicating undernutrition risk. Data were reported using confidence intervals. **Results**: In 2023, nearly 1 in 5 NSOAAP respondents were at undernutrition risk; 9.9% (95% CI 7.3–13.0%) of congregate meal participants, 20.8% (95% CI 18.3–23.5%) of home-delivered meal participants, and 21.3% (95% CI 16.7–26.4%) of participants in OAA non-nutrition programs (transportation, case management, or homemaker services). Participants in different OAA program types also differed in many demographic and health-related characteristics. **Conclusions:** Since undernutrition risk is neither a definitive diagnosis of undernutrition nor its causes, it must be followed up by further nutrition assessment.

## 1. Introduction

The Older Americans Act (OAA) of 1965 was passed by the United States (U.S.) Congress during the Johnson administration. It remains the primary federal program in the U.S. for delivery of nutrition and social services to help community dwelling adults aged 60 years and older maintain their independence. The OAA authorized a wide array of community-based health and health-related social service programs designed to support older adults in maintaining their independence, health, and quality of life. These included supportive services such as nutrition programs, housekeeping, transportation, case management, personal care, family caregiver support, and services to prevent abuse, neglect, and exploitation of older people [1]. The OAA’s aim is to target older adults with the greatest social and economic need, including individuals with low incomes, minority populations, rural residents, and those at risk of institutionalization [2].

This article’s objective is to provide prevalence estimates of undernutrition risk among a nationally representative sample of participants in a large OAA program in the U.S. using newly available post-Covid 19 data collected in 2023.

Today, the OAA Nutrition Services Program’s coverage is massive and nationwide, providing home-delivered meals (commonly called “Meals on Wheels”), congregate meals, and other nutrition-related services to roughly 2.7 million older adults annually through at least 5000 local providers throughout the U.S. Unlike many public assistance programs, eligibility for key OAA nutrition programs and services—such as congregate meals and home-delivered meals—is not based on a financial means test but is open to all adults aged 60 and older (and their spouses of any age), with voluntary financial contributions encouraged. This structure allows the OAA services to reach a broad population, recognizing that malnutrition risk and vulnerability extend beyond income alone. The OAA goals of reducing malnutrition, food insecurity, and hunger, along with promoting socialization and wellness among community-dwelling older adults, have been endorsed by health authorities nationally [3].

Participants in the various OAA nutrition programs differ in many demographic and other characteristics. Since there are substantial costs for the home-delivered meal infrastructure, home-delivered meal program participation prioritizes individuals who are homebound, have limited mobility due to poor functional status, or face significant health or social barriers that preclude attendance at congregate dining locations [4]. In contrast, participation in congregate meals is self-targeted and accessible to all of those who can attend meal sites. Non-nutrition services address participants needing specific types of assistance (e.g., with transportation, case management, or homemaker assistance with performing household chores).

Only recently have prevalence estimates for undernutrition risk in OAA programs in the U.S. for older adults living in the community become available, and definitive diagnoses of undernutrition among a representative sample of participants in them are still lacking. However, there are a good deal of data on undernutrition among community-living individuals in other countries. For example, a 2022 systematic review and meta-analysis summarized the prevalence of undernutrition, sarcopenia, and frailty among community-dwelling adults aged 50 and older in 37 studies among 100,000 combined participants in several countries [5]. It found a 17% weighted pooled prevalence estimate of undernutrition, with higher prevalence among women, and a high overlap of undernutrition with sarcopenia and frailty. Estimates of the prevalence of undernutrition in community-living older adults are available in several European countries through the European Union’s MaNuEL project, as well as more recent estimates in many countries, including the Netherlands, Poland, and Spain [6,7,8,9,10].

In contrast to these countries, while malnutrition and undernutrition in older adults are recognized as the problem in the United States [11], no large-scale studies of its prevalence in this population is available. However, the prevalence of undernutrition and its associations with lengths of hospital stay associated with malnutrition has been recently studied using the broad International Classification of Diseases (ICD) 10 diagnostic criteria for it [12]. Protein–energy malnutrition was most common (about two thirds of all cases), followed by weight loss (18%), and underweight, postsurgical non-absorption, cachexia, and nutritional neglect constituting the rest [12]. Other recent U.S.-based studies, also using ICD 10 criteria for undernutrition, examined undernutrition-related deaths in the general population. Undernutrition prevalence reported on death certificates increased with advancing age, other demographic characteristics such as race/ethnicity, and income, and varied by care site and time of the study [13,14,15]. These data, combined with the demographics and known risks of individuals post hospital discharge who are living the community, make it likely that the prevalence of undernutrition risk among community-living older adults is substantial.

The prevalence of undernutrition among community-living older Americans in OAA programs has received little attention compared to the many studies reporting the prevalence of overweight and obesity among them. However, undernutrition deserves study because it can result in weight and muscle loss, as well as poor response to injury and disease, compromised functional status, quality of life, and other clinical outcomes, ultimately having an impact on their inability to live independently in the community [16]. Recognition of the problem of undernutrition and its timely treatment has been identified as a global challenge and in the U.S. as an opportunity to reduce risk of poor health outcomes [17]. Multiple factors can contribute to undernutrition risk. These include socioeconomic and life-style factors, as well as disease and aging processes such as sensory loss [18]. Recently published results from an analysis of U.S. older adults’ dietary consumption patterns documented nearly one third had diets of poor nutrition quality and that barriers related to mobility, food preparation, and access may impact their diet quality and nutrition [19]. OAA programs help address such issues.

In 2020, the U.S. Congress expanded the purpose of the OAA Nutrition Services Program (e.g., the Title IIIC Nutrition Services) to explicitly include reducing malnutrition [20]. Undernutrition, also referred to as protein–energy malnutrition, is the form of malnutrition included in the purpose of OAA nutrition programs [16,20].

The World Health Organization’s International Classification of Diseases-11 recently approved an updated new diagnosis code 5B72 for undernutrition in adults, reflecting the present understanding that this form of malnutrition can occur because of inadequate intake, disease with or without related inflammation, and/or a combination of both conditions. The code is based on both etiology (e.g., disease and related inflammation) and phenotype (e.g., low body mass index or weight loss) [21]. The disease-related contributions to the diagnosis of undernutrition are coded 5B72.0 for undernutrition related to acute or chronic disease, injury, or infection with moderate to severe inflammation; as 5B72.1 for undernutrition related to disease with non-discernable or low levels of inflammation; and as 5B72.2 for undernutrition related to pure starvation/hunger [21]. Consistent with that terminology, the term undernutrition risk is used in this paper to describe risk of the protein–energy form of malnutrition in older adults.

To further advance the 2020 Congressional directive of reducing malnutrition, starting in 2022, screening for risk using the Malnutrition Screening Tool (MST) was incorporated into the annual cross sectional National Survey of Older Americans Act Participants (NSOAAP) by the Administration on Community Living, which oversees OAA programs. That step provided a standardized way to assess and routinely examine undernutrition risk in a nationally representative sample of OAA program participants.

In our previous analysis of the 2022 NSOAAP data, overall prevalence of undernutrition risk was 19.5% [22]. However, the 2022 study may have disproportionately reflected the effects of the COVID-19 pandemic on these community-living participants, pandemic-related OAA program delivery changes, and additional food and nutrition supports provided to older adults at that time.

The objective of this article was to determine undernutrition risk prevalence among participants who participated in the 2023 NSOAAP survey that was fielded nearly three years after the onset of the COVID-19 pandemic and to describe variations in risk by selected demographic and health-related characteristics.

## 2. Materials and Methods

### 2.1. Study Design and Participants

Data were obtained from the 2023 NSOAAP, an annual computer-assisted, telephone-administered survey fielded since 2003 [23]. The NSOAAP assesses the five OAA programs (home-delivered meals, congregate meals, transportation, case management, and homemaker services) that are overseen by the Administration on Community Living of the U.S. Department of Health and Human Services. NSOAAP survey participants were recruited through a two-stage sampling process, which has been previously described [22]. The standard survey methodology was for a sample to be chosen from the Area Agencies on Aging that administer OAA programs locally, followed by randomly sampling participants from each selected Area Agency on Aging. The 2023 research protocol was approved by the U.S. Office of Management and Budget (OMB); the OMB Control Number for the 2023 17th NSOAAP is 0985-0023. NSOAAP participants signed an Informed Consent Form for public use of their data, and no identifiers were provided in the public data set [23]. Respondents with missing, unknown, or other non-responses were excluded from the present study, resulting in a final analytical sample of 4159 in the 2023 NSOAAP survey participants.

### 2.2. Undernutrition Risk Measurement

The 2023 NSOAAP survey was the second to include the Malnutrition Screening Tool (MST) as a measure of undernutrition risk. The MST is a simple, two-question (self-reported unintended weight loss and/or eating poorly due to poor appetite [e.g., inadequate intake]) screening tool, with the summed responses yielding scores ranging from zero to five. Scores ≥ 2 were used to indicate undernutrition risk. The tool is useful in community settings since it does not require a skilled administrator and has been validated in a community living setting [24,25]. The Academy of Nutrition and Dietetics recommends the MST as appropriate to “screen adults for malnutrition (undernutrition) regardless of their age, medical history, or setting” [26]. It is also one of several first-step screening tools for risk of malnutrition based on the Global Leadership Initiative on Malnutrition (GLIM) criteria in older adults [27].

### 2.3. Demographic and Health-Related Characteristics

Food insecurity was measured using the NSOAAP “Food Insecurity Flag” variable, which classified participants as food insecure or not food insecure using the U.S. Household Food Security Survey Module [28]. This measure and other demographic variables (e.g., sex, age, race/ethnicity, rurality, income, living alone, general health status, number of compromised Activities of Daily Living (ADLs), and number of medical conditions) were collected as part of the NSOAAP’s standardized questionnaire and self-reported by NSOAAP respondents. OAA program-related variables collected from the NSOAAP survey included duration of nutrition program participation, number of additional OAA programs participated in, and percent of daily food intake from OAA meals.

### 2.4. Statistical Analysis

All analyses accounted for the complex survey design using Taylor series linearization. Prevalence was estimated as a weighted proportion and 95% confidence intervals were constructed using the Korn and Graubard method to also account for the complex survey design [29]. The Rao–Scott Chi square test was used to test differences in undernutrition risk by program type (i.e., congregate meals, home-delivered meals and non-nutrition programs), by selected characteristics (i.e., undernutrition risk, food insecurity, use of more than one other OAA program, greater than three compromised activities of daily living, and greater than five medical conditions). Pairwise comparisons of prevalence of undernutrition risk and other characteristics between groups were conducted using SUDAAN PROC Descript. Statistical significance was defined as *p* < 0.05. All analyses used survey weights and survey design variables and were performed using SAS survey procedures (SAS Software, version 9.4, SAS Institute Inc, Cary, NC, USA) to consider the complex survey design.

## 3. Results

### 3.1. Prevalence of Undernutrition Risk by Program Type

**Figure 1:**
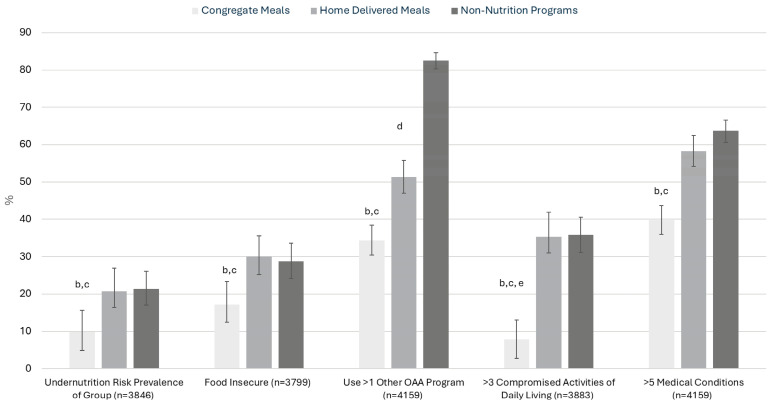
Selected reported characteristics of respondents by Older Americans Act (OAA) program type in the 2023 National Survey of Older Americans Act Participants (NSOAAP) ^a^. Abbreviations: OAA, Older Americans Act. ^a^ Sample size is unweighted and represents the total number of respondents per group. Percentages are weighted and are accompanied by 95% confidence intervals. Sample sizes vary due to missing data for some variables. ^b^ Statistically significant difference between home-delivered meal and congregate meal participants, *p* < 0.05. ^c^ Statistically significant difference between congregate meal and non-nutrition program participants, *p* < 0.05. ^d^ Statistically significant difference between home-delivered meal and non-nutrition program participants, *p* < 0.05. ^e^ Estimate does not meet National Center for Health Statistics presentation standards; see “National Center for Health Statistics Data Presentation Standards for Proportions, series 2, Number 175 August 2017” available from https://www.cdc.gov/nchs/data/series/sr_02/sr02_175.pdf (accessed on 11 May 2026).

In 2023, 20.8% (95% CI 18.3–23.5%) of home-delivered meal participants, 9.9% (95% CI 7.3–13.0%) of congregate meal participants, and 21.3% (95% CI 16.7–26.4%) of those surveyed in OAA non-nutrition programs (i.e., transportation, case management, or homemaker services) reported indications of risk for undernutrition (Figure 1). Undernutrition risk prevalence was lowest among congregate meal participants, while prevalence was similar among home-delivered meal and non-nutrition program participants.

### 3.2. Differences in Socio-Demographic Characteristics by OAA Program Type

A higher percentage of participants in the home-delivered meal and non-nutrition programs reported food insecurity, used more than one other OAA program, had greater than three compromised ADLs, and/or had greater than five medical conditions compared to congregate meal participants (Table 1). The percentage of home-delivered meal participants at undernutrition risk was higher than those participating in congregate meals among nearly all sub-groups assessed. However, among those home-delivered meal and congregate meal participants who reported fair or poor health, undernutrition risk was similar (home-delivered meals: 24.8%, 95% CI 21.2–28.8%; congregate meals: 19.5%, 95% CI 10.8–31.1%).

The home-delivered meal and non-nutrition program participants exhibited similar patterns of associations between other characteristics and prevalence of undernutrition risk. In contrast, the congregate meal and non-nutrition program participants differed from each other in many characteristics (sex, age, rurality, race/ethnicity, food security status, general health status, number of compromised ADLs, and number of medical conditions).

## 4. Discussion

The focus of this report on the prevalence of undernutrition risk is intentionally descriptive, and the analyses are limited to prevalence estimates and key subgroup comparisons rather than a comprehensive analytical study. More extensive analyses, including multivariable modeling and trend analyses, are planned for future work as additional data from 2024 become available. This article makes a timely contribution by providing the first post-pandemic national estimates of undernutrition risk in this large, federally supported OAA program serving older Americans. They are relevant to ongoing policy and programmatic efforts to address undernutrition among older adults served by that program in the United States.

In 2023, nearly one in five NSOAAP respondents overall were at undernutrition risk, as was reported in 2022 [22]. However, the actual presence of undernutrition must be confirmed by further clinical and nutritional assessment. This is necessary to evaluate the veracity of some recent reports that “malnutrition is an acutely growing public health concern in the U.S.” among older adults [13,14].

As expected, participants in the OAA’s home-delivered meal, congregate meal, and non-nutrition program types differed in many demographics, food security, health, and functional status factors as well as in the number of medical conditions they reported, indicating, as have other studies, that the different OAA program groups represent distinctive subpopulations [30,31]. The differences in undernutrition risk prevalence between OAA program groups were likely largely due to these underlying differences in health status and vulnerability, since the entry criteria, participants, and unmet needs (e.g., with transportation or performing household chores) for each program vary [32].

Other investigators and our findings indicate that the OAA programs met the goals of targeting those with greatest social and economic need, including low-income, minority, and socially isolated individuals [33,34,35]. For example, undernutrition risk was lower among congregate meal participants and higher among home-delivered meal participants and those participating in OAA non-nutrition programs. Home-delivered meal programs often prioritize individuals with limited mobility, or those who are at risk of institutionalization [35]. Services aimed at addressing undernutrition risk were more common both in this study and in the 2022 study among home-delivered meal participants—especially among those who were food insecure, had many compromised ADLs and medical conditions, and used multiple OAA services [22]. Participants in non-nutrition OAA programs in 2023 and 2022 were also found to be at higher undernutrition risk than congregate meal participants, suggesting that targeting efforts were appropriate [36]. In the 2023 study, both home-delivered meal participants and OAA non-nutrition program participants were more likely than congregate meal participants to report greater than three compromised ADLs and greater than five medical conditions.

In 2023, even among congregate meal participants, some individuals were at undernutrition risk, although overall undernutrition risk prevalence was lower among congregate meal participants than among participants in home-delivered meals and OAA non-nutrition programs. Those congregate meal participants at undernutrition risk shared some similar characteristics, such as being of Hispanic or Black race/ethnicity, reporting food insecurity, self-rated poor health, multiple limitations in ADLs, and multiple medical conditions. Future studies may identify other characteristics of these individuals contributing to decreased congregate meal participation including menu limitations, stigma and bias, and facility and location restrictions that were not examined in this study [36,37,38].

OAA non-nutrition program (e.g., transportation, case management or homemaker services) participants were also found to be at undernutrition risk, indicating that some OAA program participants who were not receiving nutrition interventions might also warrant prioritization for nutrition assessment and referral to home-delivered or congregate meal programs. Screening OAA participants in both the nutrition and non-nutrition programs could identify individuals whose undernutrition risk might otherwise have been unrecognized or “hidden”.

Undernutrition risk may arise from multiple factors including the lack of food, many chronic diseases and conditions such as sensory changes, dental issues, and other health problems affecting the elderly, as well as many other determinants of health and wellbeing. Because the Malnutrition Screening Tool used in the NSOAAP survey did not capture data other than reported unintended weight loss and appetite loss but not the underlying causes, interpretation and discussion are limited to what can be supported by the data. Further assessment is needed to identify its causes, including both lack of appropriate food and the many other factors mentioned above.

Including undernutrition risk screening in the NSOAAP may provide useful data for tracking trends in undernutrition risk, and for informing public health strategies to address the OAA nutrition program’s goal of reducing malnutrition. At the local level, undernutrition risk screening might be useful as a marker of local programmatic gaps and help in identifying ways to link those at risk to medical/social services for assessment, treatment, and amelioration of problems. To rigorously test the hypothesis that routine undernutrition risk screening within the OAA community-based programs is a useful and necessary component of comprehensive healthcare provision, analysis of data from several years of NSOAAP surveys linked to data on health outcomes would be helpful.

The OAA is the primary federal program in the U.S. for the delivery of nutrition and social services to community-dwelling older adults. Healthcare professionals and policy makers recognize its importance in promoting the health and well-being of older adults and helping them remain living in the community [39,40]. Home-delivered meal provision has been associated with reduced risk of hospital admission and higher diet quality compared to non-home-delivered meal participants, and OAA personal care services have been associated with a decreased proportion of low-care residents who would otherwise need to reside in nursing homes [41]. Participation in congregate meal programs has also been associated with higher diet quality, less food insecurity, greater socialization, and lower likelihood of hospital or nursing care facility admissions [42].

Screening for undernutrition risk in OAA community programs provides an opportunity to identify those at risk early and link them to medical and social services for further assessment, treatment, and amelioration of their nutrition problems, potentially improving health outcomes. Although it is recognized that “malnutrition is preventable” and OAA programs are “critical in identifying at-risk older adults and delivering services that help them stay nourished, independent, and engaged” [43], OAA services cannot do all of that by themselves. Strategies for dealing with those identified at undernutrition risk include comprehensive screening, referral, and coordination with available community nutrition programs to improve access to care, reduce undernutrition, and better the overall health of older adults [44]. Importantly, undernutrition risk screening through local OAA programs can address undernutrition only when its presence is confirmed by clinical assessment and followed by provision of needed nutrition, medical, and social services [45]. These tasks also require development of reciprocal links between OAA programs and medical care facilities [46]. Undernutrition risk screening through OAA programs may help facilitate these connections while also supporting the implementation of U.S. Centers for Medicare and Medicaid Services (CMS) hospital quality measures like the Malnutrition Care Score (MCS) [47] and the Age Friendly Hospital Measure, which includes a malnutrition component as part of its frailty screening [48].

This study’s strengths and limitations were like those in our prior research using the 2022 NSOAAP [22]. A strength is that this report is the first to document undernutrition risk prevalence during a non-COVID-19 pandemic year in a large nationally representative sample of OAA program participants. Other strengths are use of a validated undernutrition risk screening tool (MST) appropriate for community settings and adjustment for some potential confounding variables. This report overcomes a limitation of our earlier report on the 2022 NSOAAP that was fielded during the U.S. COVID-19 Public Health Emergency. At that time, undernutrition risk may have been affected because many older adults were isolated and ill. Further, households, states, and local programs received increased resources and waivers that impacted OAA nutrition program participants, standards and administration, and undernutrition risk.

Limitations include reporting bias in survey responses based on self-reporting. Generalization to all community-living older adults in the U.S. is not possible because the NSOAAP study population was not representative of them. Comparisons between congregate meal, home-delivered meal, and non-nutrition program groups were confounded by the substantial differences in health status and other characteristics that existed between participants in the different types of programs. The use of bivariate comparisons further limited insight into such confounding factors, and the statistical unreliability of some reported findings warrants caution in interpretation. The MST screening tool had a low threshold for indicating undernutrition risk, increasing the potential of over-identification. Indeed, further research is needed on the validity and reliability of screening tools for undernutrition risk that are suitable for application in community settings. The screening tool did not provide information on undernutrition’s causes, and it did not measure risk of other forms of malnutrition. Therefore, additional clinical nutrition status assessment would be necessary to make a definitive diagnosis of undernutrition’s presence, its causes, and presence of other forms of malnutrition or disease among the NSOAAP participants. Finally, comparisons of undernutrition prevalence between the older adults in OAA programs who were participants in the NSOAAP study to studies of community-living older adults living in other countries are difficult. The NSOAAP survey data involved only program participants living in the community, not the general population of community-living older Americans. It relied on self-reporting of undernutrition risk using a short screening tool that differed from those used in other countries. Our study included only estimates of undernutrition risk, and the survey lacked objective assessments and diagnoses to more fully assess and confirm the presence and causes of the undernutrition when it was present. Further, many of the other countries reporting prevalence estimates have national health services with stronger links between community screening and institutional health and social service facilities than those in the United States, and reporting may therefore be more complete, especially for older adults living in the community after discharge from hospital.

## 5. Conclusions

This study found that in 2023 undernutrition risk using the MST existed in community-dwelling OAA program participants, particularly among those receiving home-delivered meals and non-nutrition program services. Including the MST to monitor and track prevalence of undernutrition risk as part of the NSOAAP may help in crafting programmatic and policy recommendations to meet OAA program goals.

## Figures and Tables

**Table 1:** Undernutrition risk prevalence by Older Americans Act (OAA) program type and selected reported characteristics of respondents to the 2023 National Survey of Older Americans Act Participants (NSOAAP) ^a^.

	N	Home-Delivered Meals	N	Congregate Meals	*n*	Transportation, Home Maker, or Case Management Services	PAIRWISE: (Home-Delivered Meal Vs. Congregate Meal) *p* Value	PAIRWISE: (Home-Delivered Meal Vs. Transportation, Home Maker, or Case Management Services) *p* Value	PAIRWISE: (Congregate Meal Vs. Transportation, Homemaker, or Case Management Services) *p* Value
Total	1064	20.8 (18.3–23.5)	1222	9.9 (7.3–13.0)	1554	21.3 (16.7–26.4)	**0.0000**	0.8828	**0.0007**
Sex									
Male	395	25.3 (18.7–32.9)	404	13.8 (7.3–23)	423	21.9 (15.4–29.8)	**0.0025**	0.5355	0.1846
Female	669	18.3 (15.4–21.5)	818	7.7 (6.0–9.8)	1130	20.9 (15.5–27.2)	**0.0000**	0.3596	**0.0001**
Age group									
60–74 yrs	379	22.5 (17.6–28.0)	513	9.8 (6.8–13.5)	583	19.9 (13.1–28.3)	**0.0000**	0.5852	**0.0172**
75–84 yrs	345	20.0 (15.4–25.2)	482	10.1 (5.7–16)	582	17.7 (10.8–26.6)	**0.0029**	0.6082	0.1216
85+ yrs	337	19.6 (14.3–25.9)	231	9.8 (5.2–16.5)	391	26.9 (18.1–37.4)	**0.0269**	0.1242	**0.0052**
Rural									
No	801	20.6 (17.5–23.9)	886	10.4 (7.3–14.3)	1231	21.4 (16.4–27.2)	**0.0000**	0.8015	**0.0040**
Yes	263	22.0 (16.4–28.5)	339	8.0 (5.3–11.6)	323	20.5 (9.9–35.2)	**0.0001**	0.8277	**0.0462**
Race/ethnicity									
Hispanic	48	10.9 (2.1–29.9) ^b^	40	19.4 (0.3–74.7) ^b^	84	32.3 (7.5–68.0) ^b^	0.5756	0.1608	0.5083
White, Non-Hispanic	779	21.1 (18.3–24.1)	939	8.2 (6.3–10.5)	1043	19.8 (15.2–25.0)	**0.0000**	0.6467	**0.0001**
Black, Non-Hispanic	132	25.5 (16.8–35.9)	137	15.6 (6.1–30.4) ^b^	240	23.3 (10.5–41.1) ^b^	0.0667	0.8049	0.3877
Other	53	19.3 (4.3–46.3) ^b^	55	12.5 (2.8–31.9) ^b^	87	28.5 (9.3–55.8) ^b^	0.4150	0.5412	0.2428
Food Security Status									
Not Food-insecure	727	17.9 (15.2–20.9)	979	7.8 (6–9.8)	1089	14.8 (9.9–20.8)	0.0000	0.3167	0.0203
Food Insecure	289	25.5 (20.6–31)	209	17.3 (10.6–26)	361	36.9 (24.5–50.7)	0.0486	0.0941	0.0143
General Health									
Excellent or Very Good	151	9.4 (4.8–16.1)	418	3.8 (2.0–6.3)	248	18.4 (7.8–34.1) ^b^	0.0520	0.2063	0.0219
Good	354	19.1 (14.7–24.2)	539	10.3 (7.7–13.4)	556	19.3 (11.9–28.7)	0.0040	0.9597	0.0495
Fair or Poor	548	24.8 (21.2–28.8)	259	19.5 (10.8–31.1)	730	23.3 (16.6–31.1)	0.2187	0.6938	0.5570
>3 Compromised Activities of Daily Living (ADLs)									
Yes	322	22.9 (17.2–29.4)	103	13.0 (5.0–25.9) ^b^	401	26.6 (17.6–37.3)	0.0847	0.5095	0.0467
No	662	19.9 (16.8–23.4)	1079	9.9 (7.1–13.3)	1023	18.1 (13.5–23.6)	0.0000	0.5421	0.0110
**>5 Medical Conditions**									
Yes	623	24.0 (20.6–27.5)	520	12.8 (8.4–18.5)	969	22.7 (17.2–29)	0.0002	0.6894	0.0181
No	441	16.6 (11.9–22.3)	706	7.9 (5.5–10.9)	587	18.6 (11.6–27.6)	0.0033	0.6654	0.0191

^a^ Sample size is unweighted and represents the total number of respondents per group. Percentages are weighted and represent the proportion of the group using programs and are accompanied by 95% confidence intervals. Sample sizes vary due to missing data for some variables. ^b^ Statistically unreliable: estimate does not meet National Center for Health Statistics presentation standards; see “National Center for Health Statistics Data Presentation Standards for Proportions, Vital and Health Statistics Series 2, Number 175, August 2017 ” available from https://www.cdc.gov/nchs/data/series/sr_02/sr02_175.pdf, accessed on 11 May 2026. Note: comparisons in bold were statistically significant.

## Data Availability

Data described in the manuscript, the code book, and analytic code are available upon request to the Administration on Community Living, U.S. Department of Health and Human Services, pending application and approval by it.

## Abbreviations

The following abbreviations are used in this manuscript:

ADL	Activities of Daily Living
CI	confidence interval
MST	Malnutrition Screening Tool
NSOAAP	National Survey of Older Americans Act Participants
OAA	Older Americans Act
